# Aggregation-Sensitive Mortality Associations and Firth Penalized Logistic Regression After Hip Fracture Surgery: A Retrospective Cohort Study

**DOI:** 10.3390/jcm15187195

**Published:** 2026-09-16

**Authors:** Osman Demir

**Affiliations:** Department of Biostatistics, Faculty of Medicine, Tokat Gaziosmanpaşa University, 60100 Tokat, Türkiye; osmand.demir@gop.edu.tr

**Keywords:** hip fracture, mortality, Simpson’s paradox, aggregation-sensitive patterns, Firth logistic regression, separation, sparse data, bootstrap validation

## Abstract

**Background/Objectives:** Hip fracture mortality analyses may be influenced by aggregation-related changes in association estimates and sparse data bias, particularly for early mortality outcomes. **Methods:** This retrospective study included 297 patients who underwent hip fracture surgery between 2020 and 2025. One-year mortality was the primary outcome, and 30-day mortality was the secondary outcome. Pooled, stratum-specific, and Mantel–Haenszel odds ratios were compared to explore aggregation-sensitive changes in association estimates. Conventional maximum likelihood logistic regression and Firth penalized logistic regression were fitted using the same predictor set. The model performance was evaluated using the area under the curve (AUC), the Brier score, calibration measures, and bootstrap internal validation with 1000 resamples. **Results:** Mortality occurred in 36 of 294 evaluable patients within 30 days and 95 of 252 evaluable patients within one year. No complete reversal of the association was observed. Exploratory comparisons suggested age-related attenuation for male sex, modest masking for low hemoglobin levels across sex strata, and mild amplification for dementia and elevated NLR. In the 1-year models, age, male sex, ASA IV status, dementia or cognitive impairment, and lower preoperative hemoglobin levels were independently associated with mortality. The conventional and Firth models demonstrated nearly identical discrimination for 1-year mortality, with apparent AUC values of 0.807 and 0.806, respectively. In bootstrap validation, the conventional model yielded converged estimates in 902 of 1000 resample sets, whereas the Firth model remained estimable in all 1000 resamples. For 30-day mortality, conventional logistic regression was affected by quasi-complete separation, whereas Firth regression produced finite estimates; however, these early mortality findings were considered exploratory because only 36 deaths occurred and some estimates remained highly imprecise. **Conclusions:** Several mortality associations differed in magnitude between pooled and stratification-adjusted estimates without a complete reversal of association. Firth penalization improved estimability and resampling stability under sparse-event conditions, although it did not materially enhance the discrimination of 1-year mortality. The 30-day mortality findings should be interpreted as exploratory because of the limited number of early deaths and resulting imprecision of some estimates.

## 1. Introduction

Hip fractures are major geriatric surgical conditions associated with considerable postoperative morbidity, functional decline, institutionalization, and increased mortality [1,2,3,4]. Despite advances in surgical techniques, anesthesia, perioperative care, and rehabilitation, mortality after hip fracture surgery remains clinically important, particularly in the early postoperative period and within the first postoperative year [2,3,4,5]. Consequently, the accurate identification and interpretation of mortality-related factors are crucial for perioperative risk stratification, postoperative monitoring, and individualized care planning.

Along with advances in perioperative care, the surgical management of proximal femoral fractures has also benefited from developments in orthopedic implant design, biomaterials, and manufacturing technologies. Additive manufacturing approaches, including selective laser melting, have enabled the development of individualized femoral implants. Recent studies of 3D-printed CoCrMo components have further highlighted the importance of material processing conditions and mechanical properties for biomedical applications [6,7]. While these technological advances are important for surgical management, postoperative mortality remains strongly influenced by patient-level clinical characteristics and perioperative risk factors.

Previous research has highlighted multiple determinants of mortality following hip fracture surgery, such as advanced age, male sex, frailty, cognitive impairment, high comorbidity burden, elevated anesthetic risk, delayed operative timing, anemia, and systemic inflammatory response [5,8,9,10,11]. However, the prevalence of these prognostic variables is frequently inconsistent across clinical subgroups. Older individuals, those classified in higher American Society of Anesthesiologists physical status categories, and patients with dementia or reduced physiological reserve tended to cluster within specific risk strata. Consequently, mortality estimates obtained from the overall cohort may not accurately reflect the strength or direction of the associations within clinically meaningful subgroups.

In most observational studies of hip fracture populations, risk estimates have been reported as pooled measures for the entire cohort. While such summary estimates are informative for describing overall patterns, they can conceal trends that are specific to particular subgroups of interest. In heterogeneous clinical datasets, stratification by a key variable may reveal differences in the magnitude or, less commonly, the direction of the association between a predictor and outcome. Such discrepancies between pooled and subgroup-specific estimates are conceptually related to Simpson’s paradox, although differences in magnitude alone do not constitute a classical Simpson reversal [12,13,14]. In hip fracture surgery, these effects may be especially pertinent because age, ASA class, cognitive status, anemia, inflammatory markers, and treatment-related factors are closely interrelated in clinical practice.

The second methodological challenge in fixed-time mortality analysis is sparse data. Although long-term mortality after hip fractures is common, early outcomes, such as 30-day mortality, often involve relatively few events. Moreover, clinically important categories, including lower ASA classes or specific comorbidity subgroups, may contain a small number of patients or deaths per category. Under these conditions, conventional maximum likelihood logistic regression can yield unstable odds ratio estimates, inflated standard errors, wide confidence intervals, and complete or quasi-complete separations [15,16,17]. These issues are particularly important when the objective is not only prediction but also a clinically interpretable estimation of risk factor effects.

Firth penalized logistic regression offers a bias-reduced alternative to conventional logistic regression by adding a penalty to the likelihood function based on the Fisher information matrix [15]. This method has been shown to reduce small-sample bias and provide finite, more stable estimates in logistic regression models that are prone to separation [16,17]. Therefore, Firth penalization may be especially useful for evaluating fixed-time mortality after hip fracture surgery, where clinically meaningful subgroup analyses can produce sparse or unbalanced data.

This study aimed to examine aggregation-sensitive changes in fixed-time mortality associations after hip fracture surgery by comparing pooled and clinically stratified estimates of selected predictors. The primary outcome was 1-year mortality, and 30-day mortality was evaluated as the secondary outcome. In addition, conventional maximum likelihood logistic regression was compared with Firth penalized logistic regression to determine whether penalization yielded more stable effect estimates in sparse or separation-prone data structures. By integrating aggregation-sensitive analyses with bias-reduced regression modeling, this study aimed to enhance the statistical interpretation of mortality-related associations after hip fracture surgery.

## 2. Materials and Methods

### 2.1. Study Design and Population

This retrospective observational study included patients who underwent surgical management of hip fractures at Tokat Gaziosmanpaşa University Hospital between 2020 and 2025. The final cohort comprised 297 patients with available demographic, clinical, perioperative, laboratory, mortality, and follow-up data in the hospital records.

Patients aged ≥18 years were eligible if they had a confirmed diagnosis of hip fracture, underwent surgical treatment, and had sufficiently complete clinical and follow-up records to determine postoperative mortality status and follow-up duration. Patients were excluded if their survival status or follow-up duration could not be ascertained, if missing clinical or laboratory data precluded reliable analysis, or if the available records did not clearly document postoperative follow-up after the hip fracture surgery.

The date of surgery was used as the index. Baseline characteristics were extracted from preoperative evaluations, anesthesia charts, laboratory information systems, inpatient records, and electronic medical records of patients. Rather than a conventional time-to-event design, this study was conducted as a fixed-time mortality analysis. Postoperative mortality was evaluated at predefined time points, and logistic regression was used to estimate the odds ratios for mortality.

### 2.2. Definition of Fixed-Time Mortality Outcomes

The primary outcome was 1-year all-cause mortality, defined as death within 365 days after hip fracture surgery. The secondary outcome was 30-day all-cause mortality, defined as death within 30 days of surgery. Ninety-day mortality was evaluated as an intermediate postoperative outcome and summarized descriptively.

At each prespecified time point, mortality status was coded as 1 if the patient died within the corresponding postoperative interval and 0 if the patient was alive beyond that interval. Patients who died after a given time point were classified as survivors of the specific fixed-time outcome. Patients whose follow-up ended before the relevant time point without a recorded death were considered to have indeterminate outcomes and were excluded from the corresponding fixed time analysis. The predefined study endpoint was 31 December 2025. Patients who remained alive but had not yet reached the relevant fixed time threshold by that date were considered administratively censored for the corresponding outcomes.

Accordingly, a patient who died on postoperative day 120 was classified as alive for the 30-day and 90-day outcomes but as deceased for the 1-year outcome.

Among the 297 patients, fixed-time mortality status was ascertained for 294 at 30 days, 284 at 90 days, and 252 at 1 year. Of these, 36 of 294 evaluable patients (12.2%) died within 30 days, 55 of 284 (19.4%) died within 90 days, and 95 of 252 (37.7%) died within one year. A total of 3, 13, and 45 patients were administratively censored before the 30-day, 90-day, and 1-year thresholds, respectively, and were therefore excluded from the corresponding fixed-time analyses. All 45 patients who had not reached 365 days of follow-up had undergone surgery in 2025, and their recorded follow-up ended on the predefined study end date. Across the entire observation period, 129 patients died, and 168 were alive or censored at their last contact. The overall mortality during the available follow-up is summarized descriptively but was not analyzed as a fixed-time outcome because the follow-up duration varied among patients.

### 2.3. Candidate Predictors and Variable Definitions

Candidate predictors were selected based on their clinical relevance, prior evidence, and availability in the dataset [5,8,9,10,11]. The evaluated variables included age, sex, ASA class, type of anesthesia, time to surgery, delirium, dementia or cognitive impairment, coronary artery disease, chronic kidney disease, comorbidity count, preoperative hemoglobin level, platelet count, and preoperative NLR.

The primary multivariable models included age, sex, ASA class, dementia or cognitive impairment, preoperative hemoglobin level, and preoperative NLR. This restricted predictor set was selected before model fitting based on clinical relevance, prior evidence, and the number of available mortality events, particularly for the 30-day outcomes.

In the primary regression analyses, age, hemoglobin level, and NLR were treated as continuous variables. The odds ratios for age are reported per 10-year increments, and those for hemoglobin are reported per 1 g/dL decrease in hemoglobin levels. The NLR was transformed using a natural logarithm to account for its right-skewed distribution.

Continuous variables were categorized for descriptive and stratified analysis. The cut-off values used for the stratified analyses were selected a priori based on clinically interpretable categories and commonly used thresholds in the hip fracture literature, rather than being derived from the observed outcome distribution. Age categories were chosen to reflect clinically meaningful stages of older age, whereas the hemoglobin and NLR thresholds were selected to provide clinically interpretable subgroup comparisons rather than to optimize the statistical significance within the present dataset. ASA class was treated as a categorical variable, and sex, dementia or cognitive impairment, delirium, coronary artery disease, and chronic kidney disease were coded as binary variables.

### 2.4. Descriptive Statistical Analysis

Continuous variables are summarized as means ± standard deviations or medians with interquartile ranges, according to their distribution. Categorical variables are presented as frequencies and percentages.

Baseline characteristics were compared according to the 1-year mortality status. For continuous variables, independent-samples *t*-tests or Mann–Whitney U tests were used as appropriate for the analysis. For categorical variables, Pearson’s chi-square test with continuity correction or Fisher’s exact test was applied as appropriate.

These comparisons were conducted for a descriptive analysis. Univariate *p*-values were not used as automatic criteria for selecting variables for the multivariate models.

### 2.5. Aggregated and Stratified Association Analysis

For selected predictor–mortality relationships, crude odds ratios were first estimated in the overall cohort. The same associations were re-estimated within clinically relevant strata defined by age, ASA class, and sex. This pooled-versus-stratified comparison was used to explore aggregation-sensitive changes in the direction and magnitude of mortality associations [12,13,14]. These comparisons were not considered evidence of a classical Simpson’s paradox or statistical effect modification.

For binary predictors and binary mortality outcomes, the pooled odds ratio was calculated as follows:

(1) $$OR_{pooled}=\frac{ad}{bc},$$

where a represents exposed patients who died, b exposed patients who survived, c unexposed patients who died, and d unexposed patients who survived, respectively.

Within stratum k, the stratum-specific odds ratio was calculated as follows:

(2) $$OR_{k}=\frac{a_{k}d_{k}}{b_{k}c_{k}},$$

where $a_{k}$, $b_{k}$, $c_{k}$, and $d_{k}$ represent the corresponding cell frequencies within stratum k.

When a common association across strata was considered clinically and statistically reasonable, the Mantel–Haenszel common odds ratio was estimated as follows:

(3) $$OR\_(MH)=\frac{\sum_{k=1}^{n}\frac{a_{k}d_{k}}{n_{k}}}{\sum_{k=1}^{n}\frac{b_{k}c_{k}}{n_{k}}},$$

where $n_{k}=a_{k}+b_{k}+c_{k}+d_{k}$ represents the total number of patients in stratum k.

The preplanned analyses that were considered sensitive to aggregation focused on the following pairs: sex and mortality, examined within age and ASA class strata; anesthesia type and mortality, stratified by ASA class; dementia or cognitive impairment and mortality, assessed within age and ASA class strata; hemoglobin and mortality, evaluated separately by sex; and NLR and mortality, analyzed within strata of age and ASA class.

The direction, magnitude, and confidence intervals of the pooled, stratum-specific, and Mantel–Haenszel estimates were compared. Differences in statistical significance alone were not considered sufficient evidence of an aggregation-sensitive pattern or statistical effect modification. The homogeneity of the stratum-specific odds ratios was evaluated using the Breslow–Day test.

### 2.6. Exploratory Classification of Aggregation-Sensitive Patterns

Aggregation-sensitive patterns were explored by comparing the direction and magnitude of pooled, stratum-specific, and Mantel–Haenszel-adjusted odds ratios (ORs) [12,13,14].

The terms attenuation, masking, and amplification were used only as exploratory descriptive labels to summarize the differences between the pooled and stratification-adjusted estimates. They were not treated as formal statistical categories or as evidence of effect modification.

For descriptive purposes, a reversal pattern was considered when the direction of the pooled association contrasted with the predominant direction of stratum-specific associations. This criterion was evaluated by comparing the signs of corresponding log odds ratios.

(4) $$sign(log(OR_{pooled}))\neq sign\left(log\left(OR_{k}\right)\right),$$

A masking pattern was considered present when the pooled odds ratio was close to 1, whereas one or more clinically relevant stratum-specific odds ratios indicated a meaningful association. Attenuation was defined as the magnitude of the pooled association being smaller than that of the corresponding stratum-specific association. Amplification was defined as a pooled association being stronger than the associations observed within the relevant strata.

The degree of aggregation-related divergence is described as follows:

(5) $$D_{agg}=log(OR_{pooled})-log\left(OR_{MH}\right),$$

Values of $D_{agg}$ close to 0 indicate limited divergence between the pooled and stratification-adjusted estimates, whereas larger absolute values indicate greater divergence between the pooled and stratification-adjusted estimates.

Classification was based on the direction and magnitude of the odds ratios, their 95% confidence intervals, consistency across strata, and clinical plausibility. A change in statistical significance alone was not considered sufficient evidence of an aggregation-sensitive pattern or an effect modification. Because multiple stratified comparisons were performed, these analyses were considered exploratory and no multiplicity-adjusted inferential claims were made; nominal *p*-values were interpreted cautiously in conjunction with effect sizes, confidence intervals, and consistency across strata.

### 2.7. Interaction and Effect-Modification Analysis

Potential effect modification was evaluated using logistic regression models that included predictor-by-stratifier interaction terms. The interaction model is specified as follows:

(6) $$logit\left(P\left(Y_{i}=1\right)\right)=\beta_{0}+\beta_{1}X_{i}+\beta_{2}Z_{i}+\beta_{3}\left(X_{i}\times Z_{i}\right),$$

where $X_{i}$ is the predictor of interest, $Z_{i}$ represents the stratifying variable, and $\beta_{3}$ represents the interaction coefficient.

The conditional effect of X when Z=0 is expressed as

(7) $$OR_{\left(X|Z=0\right)}=exp\left(\beta_{1}\right),$$

The conditional effect of X when Z=1 is expressed as

(8) $$OR_{\left(X|Z=1\right)}=\exp\left(\beta_{1}+\beta_{3}\right),$$

A statistically or clinically meaningful interaction term was interpreted as evidence that the association between the predictor and mortality differed across the levels of the stratifying variables. Interaction analyses were used to distinguish exploratory aggregation-sensitive differences from statistical effect modification across clinical subgroups. To stratify variables with more than two categories, such as age group and ASA class, interactions were modeled using the corresponding indicator variable product terms, and the overall interaction was evaluated using a joint test of all the interaction coefficients.

### 2.8. Conventional Logistic Regression

Conventional binary logistic regression with maximum likelihood estimation was applied to quantify the relationships between the prespecified predictors and fixed-time mortality outcomes. Distinct models were constructed for 1-year and 30-day mortality.

For patient i, the model is defined as follows:

(9) $$logit\left(\pi_{i}\right)=\log\left(\frac{\pi_{i}}{1-\pi_{i}}\right)=\beta_{0}+\beta_{1}X_{1i}+\beta_{2}X_{2i}+\cdots +\beta_{p}X_{pi},$$

where $\pi_{i}$ represents the probability of mortality at the relevant fixed time point, $X_{1i}$ to $X_{pi}$ represent the predictor variables, and $\beta_{0}$ to $\beta_{p}$ represent the regression coefficients.

The adjusted odds ratio for predictor j is calculated as follows:

(10) $$OR_{j}=\exp\left(\beta_{j}\right),$$

The primary multivariable model comprised age, sex, ASA class, dementia or cognitive impairment, preoperative hemoglobin concentration, and preoperative NLR. To enable direct comparison, the same set of predictors and reference categories was applied to both the conventional and Firth logistic regression models.

Complete or quasi-complete separation was evaluated using cross-tabulations; the inspection of model convergence warnings; and the detection of excessively large coefficient estimates, inflated standard errors, and very wide or nonfinite confidence intervals (CIs). Adjusted odds ratios (ORs) are presented with corresponding 95% confidence intervals (CIs) and two-sided *p*-values.

### 2.9. Firth Penalized Logistic Regression

The Firth penalized logistic regression was fitted using the same outcome definitions, predictor sets, reference categories, and analytical samples as the conventional logistic regression models. This approach modifies the standard logistic likelihood by adding a Jeffreys-prior-based penalty term, thereby reducing the first-order small-sample bias and yielding finite estimates in the presence of complete or quasi-complete separation [15,16,17].

The penalized log-likelihood is defined as follows:

(11) $$l^{*}\left(\beta \right)=l\left(\beta \right)+\frac{1}{2}\log\left|I\left(\beta \right)\right|,$$

where $l\left(\beta \right)$ represents the conventional logistic log-likelihood, $I\left(\beta \right)$ represents the Fisher information matrix, and $\left|I\left(\beta \right)\right|$ denotes its determinant.

The corresponding Firth-adjusted odds ratio for predictor j was calculated as follows:

(12) $$OR_{j,Firth}=\exp\left(\beta_{j,Firth}\right),$$

Profile penalized likelihood confidence intervals were used whenever supported by the statistical software, as Wald-type intervals can be unreliable under sparse-data conditions. Firth regression was anticipated to be particularly relevant for 30-day mortality and predictor categories with small numbers of patients or deaths.

### 2.10. Comparison of Conventional and Firth Regression Models

Conventional and Firth penalized logistic regression models were compared using the same analytic sample, outcome definition, predictor set, and reference categories, with the comparison focusing primarily on estimation stability rather than on statistical significance alone.

For predictor j, the difference between the conventional maximum likelihood coefficient and the Firth coefficient is defined as follows:

(13) $$\Delta \beta_{j}=\beta_{j,ML}-\beta_{j,Firth},$$

The corresponding ratio of the odds ratios was defined as follows:

(14) $$\Delta R_{j}=\frac{OR_{j,Firth}}{OR_{j,ML}},$$

Values of $\Delta \beta_{j}$ close to 0 and $R_{j}$ close to 1 indicate similar estimates between the two methods. Larger deviations suggest a greater sensitivity of the conventional maximum likelihood estimate to sparse data or separations.

The two approaches were also compared with respect to the coefficient magnitude, odds ratio magnitude, width and finiteness of the 95% confidence intervals, convergence behavior, occurrence of complete or quasi-complete separation, and extremity of the predicted probabilities.

Because the true population parameters were unknown, empirical bias could not be directly assessed using the clinical dataset. Therefore, Firth estimates were interpreted as more stable when they remained finite, were less extreme, and were less sensitive to sparse predictor–outcome combinations than the corresponding maximum likelihood estimates.

### 2.11. Model Performance and Calibration

Model discrimination was assessed using the area under the receiver operating characteristic curve (AUC), which was interpreted as the probability that a randomly selected patient who died had a higher predicted mortality risk than that of a randomly selected patient who survived.

The overall probabilistic accuracy was evaluated using the Brier score as follows:

(15) $$Brier=\frac{1}{n}\sum_{i=1}^{n}{\left(Y_{i}-{\hat{\pi }}_{i}\right)}^{2},$$

where $Y_{i}$ represents the observed mortality outcome, ${\hat{\pi }}_{i}$ represents the predicted probability of mortality, and n represents the number of patients included in the analysis. Lower Brier scores indicate better agreement between the observed and predicted outcomes.

Calibration was assessed using the calibration intercept and slope. The calibration model is specified as follows:

(16) $$logit\left(P\left(Y_{i}=1\right)\right)=\alpha +\gamma \;logit\left({\hat{\pi }}_{i}\right),$$

where α represents the calibration intercept, and γ represents the calibration slope. Ideal calibration corresponds to α=0 and γ=1.

Differences in AUC were interpreted cautiously, as models containing the same predictors may exhibit similar discrimination despite meaningful differences in the coefficient stability. Because of the moderate sample size, the model performance was internally validated using bootstrap resampling rather than a single random training–test split. Optimism-corrected estimates of discrimination, Brier scores, and calibration slopes are reported, and the apparent calibration intercepts were calculated for the fitted models.

As a sensitivity analysis for incomplete 1-year follow-up, a Cox proportional hazards regression was performed using all 297 patients. Patients who remained alive at the end of their available follow-up were censored at their last observed follow-up time, with administrative censoring applied on 31 December 2025. The model included the same prespecified predictors as the primary 1-year logistic regression model: age, sex, ASA class, dementia or cognitive impairment, preoperative hemoglobin concentration, and the log-transformed NLR. Hazard ratios (HRs) with 95% confidence intervals are reported.

### 2.12. Missing Data

The amount and pattern of missing data were examined separately for each candidate predictor and the fixed-time mortality outcome. No missing values were identified for the predictors included in the primary multivariable models.

Patients whose mortality status could not be determined at a given time point because follow-up ended before the corresponding threshold without a recorded death were excluded only from the relevant fixed-time analysis. These observations reflect administrative censoring at the predefined study end date rather than missing predictor data or undocumented loss to follow-up. For example, a patient with less than 365 days of follow-up and no recorded death was excluded from the 1-year mortality analysis but could remain eligible for the 30- or 90-day analyses if sufficient follow-up data were available. Because the exclusion of administratively censored patients from the fixed-time logistic regression could have introduced selection bias, an additional censoring-aware time-to-event sensitivity analysis was performed for 1-year mortality.

No statistical imputations were performed. For each model, the final analytic sample size is presented alongside the number of observations that were excluded and the corresponding reasons for their exclusion.

### 2.13. Statistical Software

All analyses were conducted using Python 3.13.5. Data management and numerical computations were performed using the Pandas 2.2.3 and NumPy 2.3.5 libraries. Descriptive and inferential statistical analyses were performed using SciPy and Statsmodels, whereas receiver operating characteristic analyses, Brier score calculations, and resampling procedures were implemented using the Scikit-learn library.

Firth penalized logistic regression was implemented using a dedicated bias-reduced logistic regression routine that estimated penalized coefficients and profile penalized likelihood confidence intervals (CIs). Internal validation was performed using bootstrap resampling with 1000 replicates and a fixed random seed of 42. Conventional logistic regression models allow a maximum of 200 iterations per resample. A bootstrap fit was classified as valid only if the optimization algorithm reported convergence and all coefficient estimates were finite.

All hypothesis tests were two-sided, with *p*-values < 0.05 regarded as statistically significant. Nonetheless, aggregation-sensitive patterns were primarily evaluated according to the direction and size of the odds ratios, associated confidence intervals, consistency across strata, and clinical plausibility, rather than statistical significance alone.

Generative artificial intelligence was used during the manuscript’s preparation solely to assist with its organization and linguistic refinement. The AI tool was not used to generate or modify the original clinical data, perform statistical analyses, prepare or present statistical methods, create tables or equations, compile references, or independently determine the study results. All the manuscript content and interpretations were reviewed and verified by the author. The completed STROBE checklist is provided as Supplementary File S1.

### 2.14. Ethical Approval

The study was conducted in accordance with the principles of the Declaration of Helsinki and was approved by the Non-Interventional Scientific Research Ethics Committee of Tokat Gaziosmanpaşa University (approval no. 26-MOBAEK-306; approval date: 1 July 2026). As the study was retrospective and based on existing medical records, no additional diagnostic or therapeutic procedures were performed for this research. The requirement for informed consent was waived by the Non-Interventional Scientific Research Ethics Committee of Tokat Gaziosmanpaşa University because of the study’s retrospective design and the use of existing medical records.

## 3. Results

### 3.1. Patient Characteristics

The primary 1-year mortality analysis was restricted to 252 patients with known survival statuses at 365 days. Among them, 95 patients (37.7%) died within 1 year, and 157 (62.3%) were alive beyond 1 year. An additional 45 patients were administratively censored alive before 365 days because they had not reached the 1-year threshold by the predefined study end date of 31 December 2025, and were therefore excluded from the primary fixed-time analysis. Baseline characteristics were compared between patients with determinable 1-year mortality status and those administratively censored before 365 days. Compared with patients with determinable 1-year mortality status, administratively censored patients were younger [median age 78.0 years (IQR: 68.0–87.0) vs. 83.5 years (IQR: 76.0–89.0); *p* = 0.038], were more frequently male (60.0% vs. 42.9%; *p* = 0.049), and had higher preoperative NLR [median 7.67 (IQR: 4.97–13.02) vs. 6.15 (IQR: 3.68–10.35); *p* = 0.027]. No statistically significant differences in preoperative hemoglobin levels (*p* = 0.317), ASA class distribution (*p* = 0.719), or dementia/cognitive impairment (*p* = 0.422) were observed.

Patients who died within 1 year were older than those who survived longer [median age 87.0 years (IQR: 81.5–92.0) vs. 80.0 years (IQR: 72.0–87.0); *p* < 0.001]. The time to surgery was also greater in patients who died [2.0 days (IQR: 2.0–4.0) vs. 2.0 days (IQR: 1.0–3.0); *p* = 0.031].

The preoperative hemoglobin concentrations were lower among patients who died within 1 year than among survivors [10.7 g/dL (IQR: 10.0–12.0) vs. 11.5 g/dL (IQR: 10.5–12.6); *p* = 0.001]. The mortality group also had reduced platelet counts [200 × 10^3^/µL (IQR: 157.5–236.5) vs. 226 × 10^3^/µL (IQR: 185.0–276.0); *p* < 0.001] and higher preoperative NLR values [7.13 (IQR: 4.54–11.26) vs. 5.29 (IQR: 3.18–9.71); *p* = 0.009].

The ASA class distribution varied significantly according to the 1-year mortality status (*p* < 0.001). No deaths occurred among patients classified as ASA I, whereas ASA IV patients were overrepresented in the mortality group. Dementia or cognitive impairment was also more common in patients who died within 1 year (28.4% vs. 6.4%; *p* < 0.001).

There were no statistically significant differences between groups in sex (*p* = 0.129), type of anesthesia (*p* = 0.320), delirium (*p* = 0.250), coronary artery disease (*p* = 0.213), or chronic kidney disease (*p* = 0.421). The comorbidity count differed modestly between groups despite identical median values [2.0 (IQR: 1.0–3.0) in both groups; *p* = 0.035]. The detailed baseline characteristics are summarized in Table 1.

### 3.2. Fixed-Time Mortality Outcomes

Fixed-time mortality status was ascertained for 294 patients at 30 days, 284 patients at 90 days, and 252 patients at one year. The decreasing number of evaluable patients over time reflected censoring before the corresponding follow-up threshold.

Thirty-six of the 294 evaluable patients died within 30 days, corresponding to a 30-day mortality rate of 12.2%. At 90 days, 55 of the 284 patients had died (19.4%). The primary outcome, 1-year mortality, occurred in 95 of the 252 evaluable patients (37.7%).

Three patients were administratively censored alive before 30 days, 13 before 90 days, and 45 before 365 days because the corresponding fixed time thresholds had not been reached by the end of the study period. Over the entire follow-up period, 129 of 297 patients (43.4%) died, and 168 (56.6%) were alive or censored at their last contact. Because the follow-up duration was not uniform, overall mortality is reported descriptively and was not modeled as a fixed time endpoint (Table 2).

### 3.3. Aggregated and Stratified Associations

For the aggregation-sensitive analyses, age was categorized as <80, 80–89, and ≥90 years; ASA class as I–II, III, and IV; preoperative hemoglobin as <10 versus ≥10 g/dL; and preoperative NLR as ≥5 versus <5. Anesthesia was classified as general or non-general, with combined and spinal anesthesia grouped into the non-general category.

In the pooled analysis, male sex was associated with higher odds of 1-year mortality, although the confidence interval included the null value (OR = 1.54, 95% CI: 0.92–2.58). After stratification by age, the association was more pronounced among patients aged 80–89 years (OR = 2.71, 95% CI: 1.20–6.09), whereas no association was observed among those aged ≥90 years (OR = 0.97, 95% CI: 0.30–3.13). The age-adjusted Mantel–Haenszel estimate was OR_MH = 2.04 (95% CI: 1.16–3.57), indicating that pooling across age groups attenuated the age-stratified association. There was no statistically significant heterogeneity among the age-specific odds ratios (*p* = 0.350).

Dementia or cognitive impairment demonstrated a strong overall association with 1-year mortality (OR = 5.84, 95% CI: 2.67–12.74). This increased risk was maintained across all age groups, yielding an age-adjusted Mantel–Haenszel OR of 4.06 (95% CI: 1.79–9.20). A similarly elevated association was observed after adjustment for ASA class, with a common OR of 5.14 (95% CI: 2.25–11.74). No statistically significant heterogeneity was found across age (*p* = 0.922) or ASA strata (*p* = 0.818).

In the overall (pooled) analysis, general anesthesia was associated with reduced odds of 1-year mortality, although this association did not reach statistical significance (OR = 0.70, 95% CI: 0.42–1.17). The ASA-adjusted Mantel–Haenszel odds ratio was comparable (OR_MH = 0.68, 95% CI: 0.39–1.19), and there was no evidence of heterogeneity across ASA categories (*p* = 0.339).

Preoperative hemoglobin < 10 g/dL was associated with higher odds of 1-year mortality in the pooled analysis (OR = 1.84, 95% CI: 0.94–3.58). After sex stratification, the common odds ratio increased to 2.07 (95% CI: 1.04–4.11), suggesting a modest masking of the association in the unstratified cohort. There was no evidence that the association differed between men and women (heterogeneity, *p* = 0.734).

A preoperative NLR ≥ 5 was associated with higher odds of 1-year mortality in the pooled analysis (OR = 2.19, 95% CI: 1.27–3.78). The corresponding Mantel–Haenszel estimates were similar after age (OR_MH = 1.83, 95% CI: 1.04–3.24) and ASA stratification (OR_MH = 2.00, 95% CI: 1.13–3.53). The direction of the association was consistent across most strata, and no significant heterogeneity was observed across strata (Table 3).

### 3.4. Exploratory Aggregation-Sensitive Patterns

No complete reversal of association was observed in the main 1-year mortality analysis. None of the evaluated associations consistently shifted from higher odds in the pooled estimate to lower odds within clinically relevant strata or vice versa. However, several differences in effect magnitude were observed between the pooled and stratification-adjusted estimates. For descriptive purposes, these differences are summarized as attenuation, masking, or amplification patterns and interpreted as exploratory rather than as evidence of statistical effect modification.

The clearest attenuation pattern was observed in males. In the pooled analysis, male sex was associated with an OR of 1.54 for 1-year mortality, whereas the age-adjusted Mantel–Haenszel estimate was higher (2.04). The aggregation divergence measure is calculated as follows:

(17) $$D_{agg}=\log\left(1.54\right)-\log\left(2.04\right)=-0.279,$$

A negative value indicates that aggregation shifted the association closer to the null. This difference was most apparent among patients aged 80–89 years, in whom the male-associated mortality odds were higher, whereas the association was close to null among those aged ≥90 years. Consequently, this result was interpreted as age-related attenuation rather than a complete reversal.

Preoperative hemoglobin levels < 10 g/dL showed a modest masking pattern. The pooled association did not reach conventional statistical significance (OR = 1.84, 95% CI: 0.94–3.58), whereas the sex-adjusted Mantel–Haenszel estimate was larger and statistically distinguishable from the null (OR_MH = 2.07, 95% CI: 1.04–4.11). The corresponding divergence is

(18) $$D_{agg}=\log\left(1.84\right)-\log\left(2.07\right)=-0.119,$$

The pooled estimate was therefore modestly closer to the null than the sex-adjusted estimate, a difference that was descriptively characterized as masking or attenuation.

In contrast, the pooled association of dementia or cognitive impairment was stronger than the corresponding age-adjusted estimates. The pooled OR was 5.84, whereas the age-adjusted Mantel–Haenszel OR was 4.06, yielding the following:

(19) $$D_{agg}=\log\left(5.84\right)-\log\left(4.06\right)=0.364,$$

This difference was characterized descriptively as modest amplification. However, dementia or cognitive impairment remained strongly associated with mortality in both pooled and stratified analyses, indicating that the pooled and stratification-adjusted estimates differed in magnitude rather than in direction.

For preoperative NLR ≥ 5, the pooled OR of 2.19 was modestly higher than the age-adjusted OR_MH of 1.83 and ASA-adjusted OR_MH of 2.00. These differences indicated mild amplification in the pooled analysis; however, the direction of the association was consistent across strata.

General anesthesia showed little difference between pooled and ASA-adjusted estimates. The pooled OR of 0.70 and ASA-adjusted OR_MH of 0.68 were nearly identical, with $D_{agg}$ = 0.022. Accordingly, this association was characterized as stable across pooled and stratification-adjusted analyses (Table 4).

**Table 4:** Exploratory classification of aggregation-sensitive patterns for 1-year mortality.

Predictor	Stratifier	Pooled OR	Mantel–Haenszel OR	D_agg	Pattern Classification
Male sex	Age	1.54	2.04	−0.279	Attenuation
Male sex	ASA class	1.54	1.63	−0.059	Minimal attenuation
Dementia/cognitive impairment	Age	5.84	4.06	0.364	Amplification
Dementia/cognitive impairment	ASA class	5.84	5.14	0.127	Mild amplification
General anesthesia	ASA class	0.70	0.68	0.022	Stable/no meaningful aggregation effect
Hemoglobin < 10 g/dL	Sex	1.84	2.07	−0.119	Modest masking/attenuation
NLR ≥ 5	Age	2.19	1.83	0.177	Mild amplification
NLR ≥ 5	ASA class	2.19	2.00	0.090	Minimal amplification

**Figure 1:**
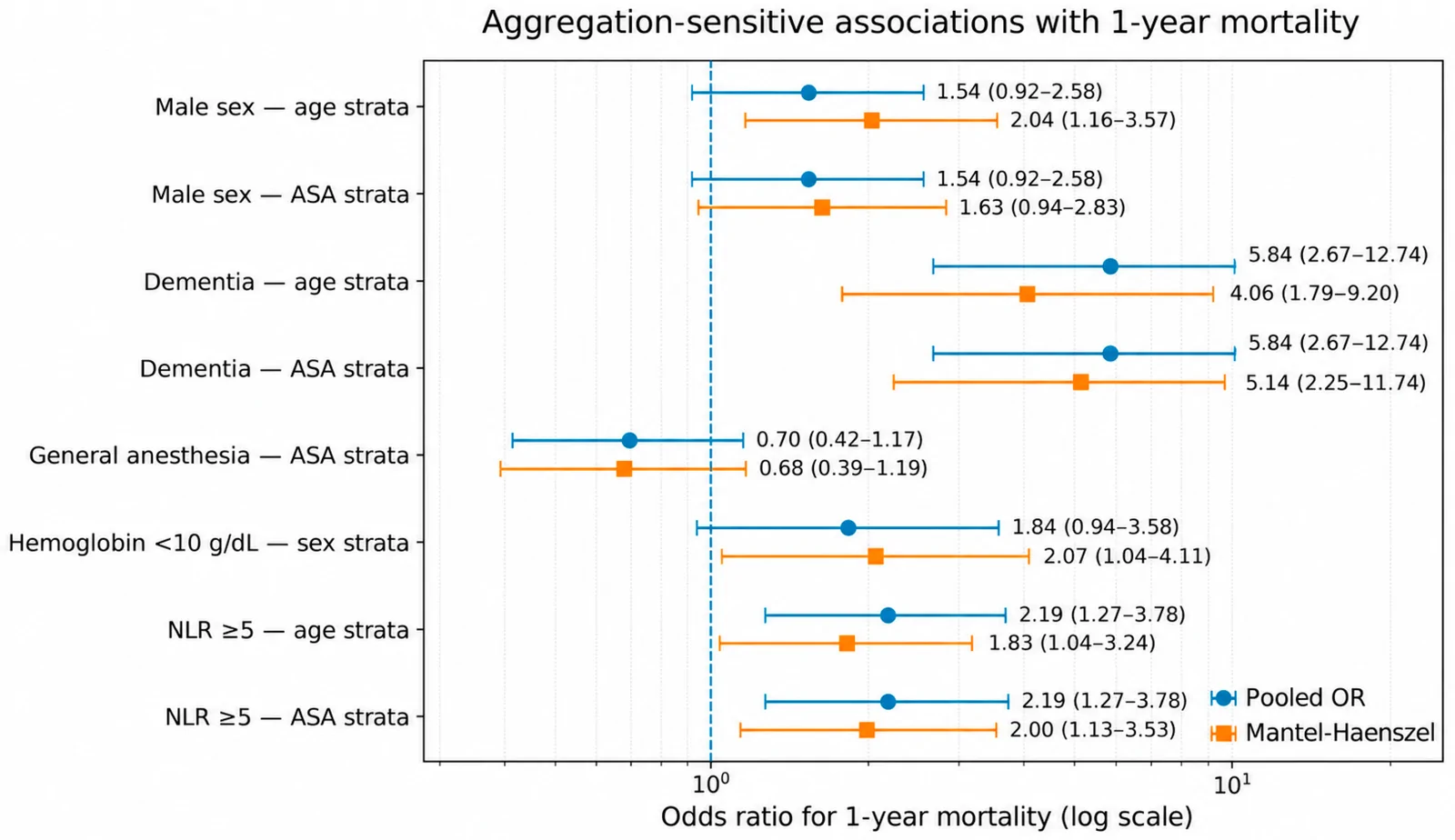
Pooled and stratification-adjusted associations with 1-year mortality after hip fracture surgery. The vertical dashed line indicates the null value (OR = 1).

Table note: $D_{agg}$ was calculated as log(OR_pooled) − log(OR_MH). Negative values indicated that the pooled estimate was closer to the null than the stratification-adjusted estimate, and positive values indicated that the pooled estimate was stronger. Pattern classification considered the effect direction, magnitude, confidence intervals, and clinical consistency. A change in statistical significance alone was not considered sufficient evidence of an aggregation-sensitive pattern. The differences between the pooled and stratification-adjusted estimates are illustrated in Figure 1. The pattern classifications are descriptive and exploratory and should not be interpreted as evidence of a classical Simpson’s paradox, confounding, or statistical effect modification.

No statistically significant predictor-by-stratifier interaction was identified in the prespecified analyses (all joint interaction *p* > 0.05). Accordingly, the differences between the pooled and stratum-specific estimates were interpreted as exploratory aggregation-sensitive differences in the effect magnitude rather than as evidence of statistical effect modification.

### 3.5. Conventional Logistic Regression Results

The conventional multivariable logistic regression model for 1-year mortality was fitted in 252 patients with known outcome status, among whom 95 died. The model converged successfully.

Older age, male sex, ASA class IV, dementia or cognitive impairment, and lower preoperative hemoglobin levels were each independently associated with increased odds of 1-year mortality. A 10-year increase in age corresponded to roughly a doubling of the odds of death within 1 year (OR = 2.01, 95% CI: 1.42–2.84, *p* < 0.001). Compared with women, men had higher adjusted odds of 1-year mortality (OR = 2.70, 95% CI: 1.43–5.13; *p* = 0.002).

Compared with ASA I–II, ASA IV was associated with a substantially higher odds of death (OR = 8.18, 95% CI: 1.47–45.52, *p* = 0.016). The odds of ASA III were also increased but estimated imprecisely and did not reach statistical significance (OR = 3.46, 95% CI: 0.69–17.33; *p* = 0.132). The wide confidence intervals for ASA categories reflected the limited number of patients and deaths in the ASA I–II reference group.

Dementia or cognitive impairment was independently related to increased 1-year mortality (OR = 3.25, 95% CI: 1.39–7.60; *p* = 0.006). Each 1 g/dL reduction in preoperative hemoglobin corresponded to a 26.9% increase in the odds of death (OR = 1.27, 95% CI: 1.06–1.52; *p* = 0.010). In contrast, the preoperative log-transformed NLR was not independently associated with 1-year mortality after adjustment for the other covariates (OR = 1.38, 95% CI: 0.92–2.07; *p* = 0.123).

The conventional 30-day mortality model included 294 evaluable patients, of whom 36 died. This model did not converge adequately and showed evidence of quasi-complete separation. Specifically, the absence or very low number of early deaths in the ASA I–II reference group led to extremely large ASA coefficients, inflated standard error, and non-informative confidence intervals. Consequently, the conventional ASA estimates for 30-day mortality were considered unstable and clinically uninterpretative. This provides the main empirical justification for using Firth penalized logistic regression (Table 5).

In the censoring-aware Cox proportional hazards sensitivity analysis including all 297 patients, older age (HR = 1.69 per 10-year increase; 95% CI: 1.33–2.17; *p* < 0.001), male sex (HR = 1.60; 95% CI: 1.06–2.42; *p* = 0.026), ASA IV status (HR = 5.73; 95% CI: 1.34–24.54; *p* = 0.019), dementia or cognitive impairment (HR = 2.18; 95% CI: 1.37–3.47; *p* = 0.001), and lower preoperative hemoglobin (HR = 1.16 per 1 g/dL decrease; 95% CI: 1.03–1.32; *p* = 0.016) were associated with higher mortality within 365 days. ASA III status (HR = 2.91; 95% CI: 0.70–12.03; *p* = 0.141) and log-transformed NLR (HR = 1.25; 95% CI: 0.95–1.65; *p* = 0.108) were not statistically significant. These findings were directionally consistent with the primary 1-year fixed-time logistic regression analysis.

### 3.6. Firth Penalized Logistic Regression Results

Firth penalized logistic regression yielded finite coefficient estimates for both fixed-time mortality outcomes in the training dataset. In contrast to the conventional 30-day model, the Firth model remained estimable despite the sparse distribution of early deaths across ASA categories.

For 1-year mortality, the Firth estimates were generally smaller than the corresponding conventional maximum likelihood estimates, whereas the overall direction of the associations remained the same. Each 10-year increase in age was associated with higher odds of death (OR = 1.93, 95% profile penalized likelihood CI: 1.40–2.77; *p* < 0.001). Male sex was also independently associated with 1-year mortality (OR = 2.61, 95% CI: 1.41–4.95; *p* = 0.002).

Compared with ASA I–II, the odds ratio for ASA III was 2.83 (95% CI: 0.75–15.80; *p* = 0.133), whereas ASA IV was associated with substantially higher mortality odds (OR = 6.49, 95% CI: 1.51–39.29; *p* = 0.010). Dementia or cognitive impairment remained independently associated with 1-year mortality (OR = 3.08, 95% CI: 1.38–7.26; *p* = 0.006).

Each 1 g/dL decrease in preoperative hemoglobin level was associated with a 25.5% increase in the odds of 1-year mortality (OR = 1.26, 95% CI: 1.06–1.51; *p* = 0.009). The log-transformed NLR was not independently associated with 1-year mortality (OR = 1.36, 95% CI: 0.92–2.04; *p* = 0.126) (Table 6).

For 30-day mortality, Firth penalization resolved the quasi-complete separation observed in the conventional model. Age remained associated with early mortality, with an OR of 1.92 per 10-year increase (95% CI: 1.20–3.27, *p* = 0.005). In the exploratory 30-day analysis, ASA IV was associated with higher mortality odds relative to ASA I–II (OR = 12.98, 95% CI: 1.49–1707.16; *p* = 0.015), although the estimate was highly imprecise, as reflected by the extremely wide confidence interval. Although the estimate remained imprecise because of the sparse reference group, it was finite and therefore clinically interpretable, unlike the conventional maximum likelihood estimate.

Male sex, ASA III status, dementia or cognitive impairment, preoperative hemoglobin level, and log-transformed NLR were not independently associated with 30-day mortality in the Firth model (Table 7).

### 3.7. Comparison of Conventional and Firth Regression Estimates

For 1-year mortality, conventional maximum likelihood and Firth penalized logistic regression yielded associations in the same direction for all prespecified predictors. Firth penalization generally reduced the magnitude of the estimated coefficients, with the largest attenuation observed for the ASA categories in which the reference group included relatively few patients who died.

For ASA IV, the conventional model estimated an adjusted OR of 8.18, whereas the Firth model yielded a smaller estimate of 6.49 for ASA IV. Similarly, the OR for ASA III decreased from 3.46 in the conventional model to 2.83 after Firth penalization. These reductions suggest that the maximum likelihood estimates are more sensitive to sparse outcome–category combinations.

For the remaining predictors, the differences between the two methods were negligible. The OR per 10-year increase in age decreased from 2.01 to 1.93, the OR for male sex from 2.70 to 2.61, and the OR for dementia or cognitive impairment from 3.25 to 3.08, respectively. The estimates for preoperative hemoglobin and log-transformed NLR were nearly unchanged between the models.

The difference between the conventional and Firth coefficients is calculated as follows:

(20) $$\Delta \beta_{j}={\hat{\beta }}_{j,ML}-{\hat{\beta }}_{j,Firth},$$

The ratio of the corresponding odds ratios was calculated as follows:

(21) $$R_{j}=\frac{OR_{j,Firth}}{OR_{j,ML}},$$

For most predictors, $R_{j}$ was close to 1, indicating similar estimates of effect size. In contrast, $R_{j}$ was 0.79 for ASA IV and 0.82 for ASA III, demonstrating a greater penalization effect in the sparse ASA categories.

The methodological differences between the models were more pronounced for 30-day mortality. Conventional maximum likelihood estimation exhibited quasi-complete separation and yielded unstable ASA coefficients with inflated standard errors and non-informative confidence intervals. Firth penalization resolved these estimation failures and produced finite coefficients for all predictors. Nonetheless, the very wide confidence intervals for ASA III and ASA IV indicate that, although penalization improved estimability, it could not compensate for the limited number of early deaths in the reference group.

**Figure 2:**
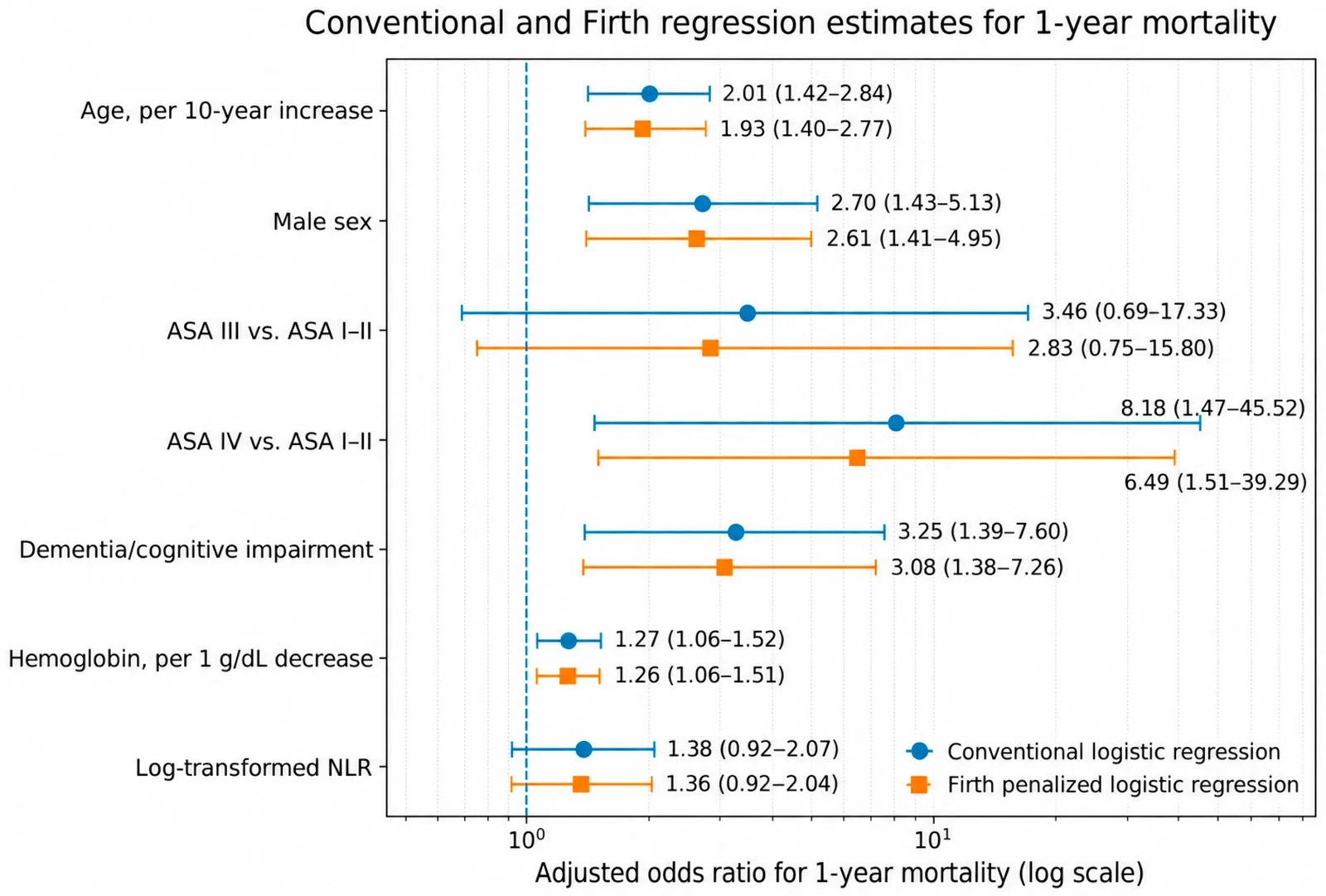
Comparison of adjusted odds ratio estimates obtained from conventional and Firth penalized logistic regression models for 1-year mortality. The vertical dashed line indicates the null value (OR = 1).

The adjusted odds ratio estimates obtained from the conventional and Firth penalized logistic regressions are compared in Figure 2 (Table 8).

### 3.8. Model Performance and Calibration Results

For 1-year mortality, conventional logistic regression and Firth penalized logistic regression demonstrated nearly identical discrimination. The apparent AUC was 0.807 for conventional logistic regression and 0.806 for Firth penalized logistic regression. The apparent Brier scores were also the same (0.171 for both the models).

After 1000 bootstrap resamples, the optimism-corrected AUCs decreased to 0.787 for the conventional logistic regression and 0.786 for the Firth model. The corresponding optimism-corrected Brier scores were 0.182 for both approaches. These results indicate that penalization did not materially affect the overall ranking or probabilistic accuracy of the 1-year mortality predictions.

The apparent calibration slopes were 1.000 for conventional logistic regression and 1.056 for the Firth model, respectively. After optimism correction, the calibration slopes decreased to 0.868 and 0.926, respectively. Thus, both models showed some overfitting during internal validation; however, the Firth model maintained a calibration slope closer to the ideal value of 1.

The apparent calibration intercept was approximately 0 for conventional logistic regression and 0.006 for the Firth model, indicating minimal systematic under- or over-prediction within the development dataset.

An additional methodological difference emerged during bootstrap validation. The conventional model yielded valid, converged estimates in 902 of the 1000 bootstrap samples, whereas the Firth model produced finite estimates in all 1000 samples. Convergence failures in the conventional model occurred mainly in resamples with sparse or separated ASA–mortality patterns. This finding supports the greater numerical stability of Firth penalization, despite the nearly identical AUC and Brier scores obtained using both methods.

For 30-day mortality, performance measures were not reported for the conventional logistic model because valid maximum-likelihood convergence was not achieved. In contrast, the Firth model remained estimable, with an apparent AUC of 0.814, Brier score of 0.091, calibration intercept of 0.034, and calibration slope of 1.082 (Table 9).

## 4. Discussion

This study evaluated fixed-time mortality after hip fracture surgery from two complementary methodological perspectives: the impact of aggregation on estimated associations and the stability of conventional versus Firth penalized logistic regression under sparse event conditions. Three principal findings were obtained. First, no complete reversal of association was identified; however, exploratory differences in the effect magnitude between pooled and stratification-adjusted estimates were observed and descriptively summarized as attenuation, masking, or amplification. Second, advanced age, male sex, ASA IV status, dementia or cognitive impairment, and lower preoperative hemoglobin levels were independently associated with 1-year mortality. Third, conventional and Firth logistic regression produced broadly comparable estimates for 1-year mortality, whereas Firth penalization provided finite and numerically more stable estimates in the exploratory 30-day mortality analysis, in which conventional maximum likelihood estimation was affected by quasi-complete separations. Overall, these results indicate that aggregation primarily altered the magnitude rather than the direction of the selected associations, and that the benefits of penalization were most evident when events and predictor–outcome combinations were sparse. The primary contribution of this study is methodological, rather than predictive. By combining aggregation-sensitive analyses with bias-reduced logistic regression, we illustrated how clinically heterogeneous subgroup structures and sparse event distributions can influence the magnitude and numerical stability of mortality estimates.

Fixed-time logistic regression provides clinically interpretable risk estimates at prespecified postoperative time points but does not use the exact timing of events and requires the exclusion of patients who have not yet reached the corresponding time threshold. In contrast, time-to-event methods account for censoring and event timing, although their effect measures address a different estimand. Accordingly, the Cox model in this study was used as a censoring-aware sensitivity analysis rather than as a replacement for the prespecified fixed-time analyses.

The observed mortality rates were 12.2%, 19.4%, and 37.7% at 30 days, 90 days, and 1 year, respectively. The mortality after hip fracture varies considerably across studies because of differences in age, frailty, prefracture functional status, comorbidity burden, cognitive impairment, institutional residence, perioperative management, and follow-up procedures [1,2,3,4,5,18]. A recent systematic review and meta-analysis identified advanced age, male sex, ASA class III or IV, dementia, renal impairment, heart failure, malignancy, and low hemoglobin as the most consistently reported preoperative predictors of early mortality [5]. Contemporary international evidence also demonstrates substantial geographic and case-mix variations in both short- and long-term mortality following hip fractures [18]. Accordingly, the relatively high 1-year mortality rate in the present cohort is likely to reflect its advanced age distribution and considerable perioperative risk burden rather than an isolated institutional effect.

Advanced age was one of the most consistent predictors in this study. In both the conventional and Firth models, each 10-year increase in age was associated with an approximately two-fold increase in the odds of 1-year mortality. This result aligns with previous meta-analyses and large observational studies that have reported a progressive increase in mortality with advancing age [2,3,4,5,19]. However, chronological age alone may not fully reflect the biological vulnerability of the patient. Frailty, which encompasses deficits in physiological reserve, cognition, mobility, nutrition, and comorbidity, may provide prognostic information beyond age [20,21,22]. Recent systematic reviews have shown that frailty is associated with increased short-term and 1-year mortality and a higher risk of postoperative complications after hip fracture surgery [21,22]. Although a formal frailty measure was not available in the present dataset, age, ASA class, dementia, and hemoglobin levels may partially reflect overlapping dimensions of physiological vulnerability.

Male sex was independently associated with 1-year mortality, with adjusted odds approximately 2.6–2.7 times those of female patients. Higher postfracture mortality in men has been consistently reported and may reflect a greater comorbidity burden, a poorer physiological reserve, more severe acute illness, and differences in prefracture functional status [2,4,5,19]. In the present study, the sex–mortality relationship was aggregation-sensitive, with a pooled OR of 1.54, and an age-adjusted Mantel–Haenszel OR of 2.04. The association was strongest among patients aged 80–89 years and was nearly absent among those aged ≥90 years. Consequently, combining age groups shifted the overall estimate toward the null and partially obscured a stronger association in an important clinical subgroup. This pattern was therefore descriptively characterized as attenuation rather than a reversal of association.

No complete reversal of association was detected in prespecified analyses. However, the lack of a change in the effect direction does not imply that aggregation has no impact on the results. Although no complete reversal was observed, the pooled and stratification-adjusted estimates differed in magnitude for several associations [12,13,14]. For descriptive purposes, these differences are summarized as attenuation, masking, or amplification patterns. These patterns were considered exploratory and should not be interpreted as formal variants of Simpson’s paradox or as evidence of statistical effect modification. In this cohort, male sex demonstrated age-related attenuation, hemoglobin levels < 10 g/dL exhibited modest masking when data were pooled across sexes, and dementia and elevated NLR showed mild amplification. These exploratory differences are relevant because a single pooled estimate may be interpreted as applying uniformly to all patients. In heterogeneous clinical datasets, differences in the distribution of age, ASA class, sex, and cognitive status across exposure groups may influence the magnitude of the pooled association estimates. Therefore, pooled and stratified estimates should be interpreted jointly by considering the effect direction, effect size, confidence intervals, consistency across strata, and clinical plausibility, rather than relying solely on changes in statistical significance [12,13,14]. Because multiple stratified comparisons were examined without formal multiplicity adjustment, these subgroup findings should be interpreted as exploratory and hypothesis-generating, rather than confirmatory.

From a statistical standpoint, the attenuation, masking, and amplification labels used in this study are descriptive summaries of differences between the pooled and stratification-adjusted estimates. Such differences may arise from the distribution of clinically important covariates across exposure groups and should not be interpreted as evidence of confounding or effect modification. Using pooled, stratum-specific, and Mantel–Haenszel estimates together yielded complementary insights: the pooled estimate captured the overall association at the cohort level, stratum-specific estimates characterized conditional relationships within subgroups, and the Mantel–Haenszel estimate quantified the association after accounting for stratification. Notably, none of the prespecified analyses identified statistically significant predictor–stratification interactions. This pattern is consistent with the view that the observed differences mainly reflect aggregation-related changes in the effect magnitude rather than providing definitive evidence of genuine statistical effect modification.

Dementia or cognitive impairment was strongly associated with 1-year mortality in the pooled, stratified, conventional, and Firth analyses. The pooled OR exceeded the age-adjusted Mantel–Haenszel estimate, a difference that was descriptively characterized as modest amplification; however, the direction of the association and its clinical interpretation remained the same. Cognitive impairment may contribute to higher mortality through impaired mobility, lower engagement in rehabilitation, malnutrition, delirium, infection, aspiration, communication barriers, and greater dependence on activities of daily living [5,9,23,24]. Recent registry-based studies and evidence syntheses have similarly reported that patients with dementia or cognitive impairment have increased mortality and poorer functional recovery after hip fractures [23,24]. The sustained association between dementia and mortality after adjustment for age, sex, ASA class, hemoglobin, and NLR indicates that cognitive impairment adds prognostic information beyond general age-related or anesthetic risks.

Lower preoperative hemoglobin levels were independent predictors of 1-year mortality. Each 1 g/dL reduction corresponded to an approximately 25–27% increase in the odds of death. In the categorical aggregation analysis, the pooled OR for hemoglobin < 10 g/dL was 1.84, whereas the sex-adjusted Mantel–Haenszel OR was 2.07. This difference was descriptively characterized as modest masking and may reflect sex-related differences in the distribution of baseline hemoglobin levels across the cohorts. Preoperative anemia may indicate chronic inflammation, renal impairment, malnutrition, occult bleeding, or reduced cardiopulmonary reserve, and may lower tolerance to perioperative blood loss and physiological stress. Prior studies have linked both preoperative and chronic pre-injury anemia to postoperative complications and higher 1-year mortality rates after hip fractures [10,25,26]. Collectively, these observations support hemoglobin as a practical risk marker, although such observational associations do not demonstrate that treating anemia in isolation will necessarily reduce mortality rates.

A preoperative NLR ≥ 5 was associated with higher 1-year mortality in pooled analyses; however, its independent association weakened after adjustment for age, sex, ASA class, dementia, and hemoglobin. The pooled estimate was moderately larger than the age- and ASA-adjusted Mantel–Haenszel estimates, a difference that was descriptively characterized as a mild amplification. The NLR integrates neutrophil-dominant inflammatory activity and lymphocyte-related immune reserve and has been proposed as a readily available prognostic marker for hip fractures [11,27]. However, threshold definitions, measurement time points, follow-up periods, and adjustment sets have differed considerably among studies. Our findings suggest that NLR may reflect global inflammatory and physiological burden but may provide limited independent prognostic information once major clinical predictors are considered.

General anesthesia showed no significant association with 1-year mortality, and the pooled and ASA-adjusted estimates were almost identical. This consistency indicates that stratification by ASA class did not meaningfully modify the anesthesia–mortality relationship in this cohort. Prior systematic reviews comparing general and regional anesthesia have yielded heterogeneous and largely inconclusive results regarding mortality [28,29]. Interpretation is further complicated by confounding by indication, as the choice of anesthesia is shaped by factors such as anticoagulation status, hemodynamic stability, fracture and surgical characteristics, clinician preferences, and institutional protocols. Accordingly, our results should not be viewed as evidence that the techniques are equivalent but rather as indicating no clear independent association between anesthesia type and mortality within this dataset.

The ASA class has emerged as one of the strongest clinical predictors of postoperative mortality. Compared with ASA I–II status, ASA IV status was associated with substantially higher 1-year mortality in both the conventional and Firth models. However, the confidence intervals were wide because few deaths occurred in the lower ASA reference group. This sparse reference structure was particularly problematic for 30-day mortality, where the conventional model yielded extremely large ASA coefficients, inflated standard errors, and confidence intervals that were not clinically informative. The observed association between higher ASA class and early mortality aligns with contemporary cohort and systematic review findings [5,19]. Nonetheless, the ASA class is best viewed as a broad marker of systemic disease burden and physiological reserve rather than as a discrete causal factor.

The comparison between conventional and Firth regression illustrates the contrast between estimation under adequate information and sparse-data conditions. For 1-year mortality, 95 events were available, and both methods produced similar coefficient directions and effect sizes. The Firth estimates were slightly smaller, particularly for ASA III and ASA IV, indicating a shrinkage of the most unstable maximum likelihood estimates. The differences between the two methods for age, sex, dementia, hemoglobin, and NLR were minimal. Thus, when the outcome was sufficiently frequent and separation was limited, penalization did not materially change the clinical conclusions.

The situation differed for 30-day mortality, for which only 36 deaths were observed in this study. Conventional maximum likelihood estimation was affected by quasi-complete separation, mainly because few or no early deaths occurred in some ASA reference category combinations. Under these conditions, the maximum likelihood coefficients can become extremely large, the standard errors can be inflated, and the confidence intervals can be unbounded or clinically uninterpretable [15,16,17,30]. Firth penalization yielded finite coefficients and profile-penalized likelihood confidence intervals for all predictors, underscoring its practical value in separation-prone analyses. However, the very wide confidence intervals for ASA III and ASA IV show that penalization improved estimability without closing the underlying information gap. Therefore, finite estimates should not be mistaken for accurate estimates. Accordingly, the 30-day mortality findings should be regarded as exploratory and interpreted cautiously because of the limited number of early deaths and resulting imprecision of some estimates.

The conventional and Firth models had almost identical apparent and optimism-corrected AUC and Brier scores for 1-year mortality, as expected, because both contained the same predictors and generated similar rankings. However, comparable discrimination and overall accuracy do not guarantee equivalent numerical behaviors. During bootstrap internal validation, the conventional model produced valid, converged estimates in 902 of 1000 resamples, whereas the Firth model yielded finite estimates in all 1000 resamples. The optimism-corrected calibration slope was also closer to the ideal value of 1 in the Firth model. Thus, the main advantage of penalization is expressed through finite estimation, coefficient stability, computational robustness, and resampling reproducibility rather than through improved rank discrimination. These findings support the view that penalization can enhance numerical stability and reduce small-sample coefficient bias, even when conventional measures of predictive performance remain nearly unchanged [15,16,17,30,31,32,33]. Nonetheless, Firth estimates may still produce biased predicted probabilities in markedly imbalanced outcomes, underscoring the need to distinguish effect estimation from prediction [17].

These findings have clinical and methodological implications. Clinically, the interpretation of mortality-related factors after hip fracture should not rely solely on pooled associations when the distribution of age, sex, ASA class, or cognitive status may influence the magnitude of pooled estimates. Stratified analyses may reveal clinically relevant differences in effect magnitude, even when no reversal of the association is observed. Methodologically, the choice between conventional and penalized logistic regression should be guided by the number of events, sparsity within reference categories, convergence diagnostics, coefficient magnitude, and confidence interval width, rather than by a rigid event-per-variable rule alone [30,31,32,33]. In adequately informative samples, the conventional and Firth models may lead to nearly identical conclusions. In contrast, Firth penalized logistic regression may be particularly useful when conventional maximum likelihood estimation is affected by complete or quasi-complete separation, sparse outcome events, very small cell counts, nonconvergence, or unstable or nonfinite coefficient estimates.

This study had several strengths. Mortality was assessed at clearly defined postoperative time points, and patients with indeterminate outcomes at a given threshold were excluded from fixed-time analysis. Pooled, stratum-specific, and Mantel–Haenszel estimates were examined jointly, rather than characterizing aggregation-sensitive differences solely based on changes in *p*-values. Conventional and Firth models were fitted using the same analytical samples, predictors, coding, and reference categories to enable direct methodological comparison. In addition, bootstrap validation revealed convergence instability that would not have been evident from a single-model fit.

This study had some limitations. First, its retrospective, single-center design and modest sample size may restrict the generalizability of the findings. In addition, 45 patients did not reach 365 days of follow-up by the predefined study end date and were therefore excluded from the primary 1-year fixed-time analysis. These administratively censored patients differed from those with determinable 1-year mortality status in terms of age, sex, and preoperative NLR, indicating a potential for selection bias in the fixed-time analysis. However, a censoring-aware Cox proportional hazards sensitivity analysis, including all 297 patients, yielded findings that were directionally consistent with the primary 1-year logistic regression model. Second, only 36 deaths occurred within 30 days; therefore, the 30-day analyses were considered exploratory, and some ASA-specific estimates remained markedly imprecise despite the Firth penalization. Multiple stratified comparisons were performed without formal adjustment for multiplicity; therefore, these subgroup findings should be interpreted as exploratory and hypothesis-generating. Third, continuous predictors were grouped for stratified analyses to create clinically meaningful patient subgroups. Although the main regression models retained these predictors in a continuous form, categorization likely reduced the available information and introduced dependence on arbitrary thresholds. Fourth, residual confounding cannot be ruled out because direct measures of frailty, pre-fracture functional status, nutritional status, living situation, and rehabilitation intensity were not available. Frailty and early postoperative mobilization have been linked to mortality and other adverse outcomes after hip fractures and may therefore represent important unmeasured prognostic variables [21,22,34]. The direction and magnitude of any bias arising from these unmeasured factors cannot be determined using the present data. Fifth, multiple aggregation-sensitive comparisons were undertaken; consequently, attenuation, masking, and amplification patterns should be regarded as structured exploratory observations rather than definitive evidence of causal effect modification. Sixth, the model performance was assessed using internal bootstrap validation only, and external validation is needed before these models can be used for individualized clinical prediction. Finally, although logistic regression provides clinically interpretable estimates at prespecified time points, it does not exploit the full information contained in event times and should not be viewed as a replacement for time-to-event modeling.

## 5. Conclusions

In this cohort of patients undergoing hip fracture surgery, several mortality associations differed in magnitude between pooled and stratification-adjusted estimates; however, no complete reversal of association or statistically significant effect modification was detected. The association with male sex showed age-related attenuation, low hemoglobin levels exhibited modest masking across sex strata, and dementia and elevated NLR demonstrated mild amplification. Collectively, these results indicate that pooled estimates may only partially capture clinically meaningful subgroup patterns, even when the direction of the association is consistent with our hypothesis.

Conventional and Firth logistic regression produced closely aligned estimates for 1-year mortality, whereas Firth penalization yielded substantially greater numerical stability for 30-day mortality under sparse-event and quasi-separation conditions. The main advantage of Firth regression in this setting is enhanced estimability and resampling stability rather than improved discrimination. Combining aggregation-sensitive analyses with penalized logistic regression may strengthen the reliability and clinical interpretability of fixed-time mortality studies after hip fracture surgery.

## Figures and Tables

**Table 1:** Baseline characteristics according to 1-year mortality status.

Variable	Survived Beyond 1 Year (*n* = 157)	Died Within 1 Year (*n* = 95)	*p*-Value
Age, years	80.0 (72.0–87.0)	87.0 (81.5–92.0)	<0.001
Sex			0.129
Male	61 (38.9)	47 (49.5)	
Female	96 (61.1)	48 (50.5)	
ASA class			<0.001
ASA I	7 (4.5)	0 (0.0)	
ASA II	23 (14.6)	2 (2.1)	
ASA III	112 (71.3)	65 (68.4)	
ASA IV	15 (9.6)	28 (29.5)	
Type of anesthesia			0.320
General	75 (47.8)	37 (38.9)	
Combined	13 (8.3)	7 (7.4)	
Spinal	69 (43.9)	51 (53.7)	
Time to surgery, days	2.0 (1.0–3.0)	2.0 (2.0–4.0)	0.031
Delirium	9 (5.7)	10 (10.5)	0.250
Dementia/cognitive impairment	10 (6.4)	27 (28.4)	<0.001
Coronary artery disease	34 (21.7)	28 (29.5)	0.213
Chronic kidney disease	15 (9.6)	13 (13.7)	0.421
Preoperative hemoglobin, g/dL	11.5 (10.5–12.6)	10.7 (10.0–12.0)	0.001
Preoperative platelet count, ×10^3^/µL	226.0 (185.0–276.0)	200.0 (157.5–236.5)	<0.001
Preoperative NLR	5.29 (3.18–9.71)	7.13 (4.54–11.26)	0.009
Comorbidity count	2.0 (1.0–3.0)	2.0 (1.0–3.0)	0.035

Table note: Values are presented as median (interquartile range) or *n* (%). Continuous variables were compared using the Mann–Whitney U test. Categorical variables were compared using Pearson’s chi-square test with continuity correction or Fisher’s exact test, as appropriate. The analysis included 252 patients with determinable 1-year mortality status; 45 patients who were censored alive before 365 days were excluded.

**Table 2:** Fixed-time mortality outcomes after hip fracture surgery.

Outcome	Evaluable Patients, *n*	Deaths, *n* (%)	Survived Beyond Time Point, *n* (%)	Censored Alive Before Time Point, *n*
30-day mortality	294	36 (12.2)	258 (87.8)	3
90-day mortality	284	55 (19.4)	229 (80.6)	13
1-year mortality	252	95 (37.7)	157 (62.3)	45
Overall mortality *	297	129 (43.4)	168 (56.6)	—

Table note: Percentages for fixed-time outcomes were calculated using patients whose mortality status was determined at the corresponding time points. Patients who were censored alive before the relevant threshold were excluded from the fixed-time analysis. * Overall mortality was evaluated during the entire available follow-up period and was considered exploratory because the follow-up durations differed among patients.

**Table 3:** Pooled, stratum-specific, and Mantel–Haenszel associations with 1-year mortality.

Predictor and Stratifier	Stratum	OR (95% CI)	Mantel–Haenszel OR (95% CI)	Heterogeneity *p*
Male sex—pooled	Overall	1.54 (0.92–2.58)	—	—
Male sex—age strata	<80 years	2.33 (0.80–6.85)	2.04 (1.16–3.57)	0.350
	80–89 years	2.71 (1.20–6.09)		
	≥90 years	0.97 (0.30–3.13)		
Male sex—ASA strata	ASA I–II	0.88 (0.05–15.33)	1.63 (0.94–2.83)	0.866
	ASA III	1.60 (0.86–2.99)		
	ASA IV	2.00 (0.56–7.16)		
Dementia/cognitive impairment—pooled	Overall	5.84 (2.67–12.74)	—	—
Dementia—age strata	<80 years	4.53 (0.27–76.08)	4.06 (1.79–9.20)	0.922
	80–89 years	3.57 (1.31–9.76)		
	≥90 years	5.24 (1.03–26.64)		
Dementia—ASA strata	ASA I–II	3.93 (0.12–124.06) *	5.14 (2.25–11.74)	0.818
	ASA III	4.98 (2.02–12.26)		
	ASA IV	6.63 (0.75–58.57)		
General anesthesia—pooled	Overall	0.70 (0.42–1.17)	—	—
General anesthesia—ASA strata	ASA I–II	0.14 (0.01–3.06) *	0.68 (0.39–1.19)	0.339
	ASA III	0.78 (0.42–1.46)		
	ASA IV	0.58 (0.16–2.06)		
Hemoglobin < 10 g/dL—pooled	Overall	1.84 (0.94–3.58)	—	—
Hemoglobin < 10 g/dL—sex strata	Male	2.49 (0.68–9.09)	2.07 (1.04–4.11)	0.734
	Female	1.91 (0.85–4.32)		
NLR ≥ 5—pooled	Overall	2.19 (1.27–3.78)	—	—
NLR ≥ 5—age strata	<80 years	1.32 (0.47–3.69)	1.83 (1.04–3.24)	0.438
	80–89 years	1.67 (0.72–3.83)		
	≥90 years	3.61 (1.06–12.25)		
NLR ≥ 5—ASA strata	ASA I–II	0.88 (0.05–15.33)	2.00 (1.13–3.53)	0.498
	ASA III	1.82 (0.96–3.44)		
	ASA IV	4.03 (0.99–16.35)		

Table note: ORs greater than 1 indicate higher odds of 1-year mortality. Mantel–Haenszel ORs represent common associations adjusted for stratifications. Heterogeneity *p*-values were obtained from the Breslow–Day test for homogeneity of stratum-specific odds ratios. * A continuity correction of 0.5 was used when a contingency table cell had no observations. General anesthesia was compared with combined and spinal anesthesia. The analyses included 252 patients with a known 1-year mortality status.

**Table 5:** Conventional multivariable logistic regression for 1-year mortality.

Predictor	β Coefficient	Adjusted OR (95% CI)	*p*-Value
Age, per 10-year increase	0.697	2.01 (1.42–2.84)	<0.001
Male sex	0.995	2.70 (1.43–5.13)	0.002
ASA III vs. ASA I–II	1.240	3.46 (0.69–17.33)	0.132
ASA IV vs. ASA I–II	2.101	8.18 (1.47–45.52)	0.016
Dementia/cognitive impairment	1.179	3.25 (1.39–7.60)	0.006
Hemoglobin, per 1 g/dL decrease	0.238	1.27 (1.06–1.52)	0.010
Log-transformed NLR	0.321	1.38 (0.92–2.07)	0.123

Table note: The model included 252 patients and 95 patients who died. ASA I–II, female sex, and absence of dementia or cognitive impairment were used as reference categories. Hemoglobin was coded so that the odds ratio represents a 1 g/dL decrease. The NLR was natural-log transformed. OR, odds ratio; CI, confidence interval; ASA, American Society of Anesthesiologists physical status; NLR, neutrophil–lymphocyte ratio.

**Table 6:** Firth penalized logistic regression for 1-year mortality.

Predictor	Firth β	Adjusted OR (95% Profile Likelihood CI)	*p*-Value
Age, per 10-year increase	0.659	1.93 (1.40–2.77)	<0.001
Male sex	0.960	2.61 (1.41–4.95)	0.002
ASA III vs. ASA I–II	1.040	2.83 (0.75–15.80)	0.133
ASA IV vs. ASA I–II	1.871	6.49 (1.51–39.29)	0.010
Dementia/cognitive impairment	1.124	3.08 (1.38–7.26)	0.006
Hemoglobin, per 1 g/dL decrease	0.227	1.26 (1.06–1.51)	0.009
Log-transformed NLR	0.309	1.36 (0.92–2.04)	0.126

**Table 7:** Firth penalized logistic regression for 30-day mortality.

Predictor	Firth β	Adjusted OR (95% Profile Likelihood CI)	*p*-Value
Age, per 10-year increase	0.651	1.92 (1.20–3.27)	0.005
Male sex	0.236	1.27 (0.59–2.71)	0.544
ASA III vs. ASA I–II	1.094	2.99 (0.36–389.14)	0.382
ASA IV vs. ASA I–II	2.563	12.98 (1.49–1707.16)	0.015
Dementia/cognitive impairment	0.454	1.58 (0.63–3.70)	0.317
Hemoglobin, per 1 g/dL decrease	−0.005	1.00 (0.79–1.25)	0.968
Log-transformed NLR	0.411	1.51 (0.91–2.53)	0.110

Table note: Firth penalized logistic regression was used to reduce the small-sample bias and obtain finite estimates under sparse data and separation conditions. Confidence intervals and *p*-values were derived from the profile penalized likelihood. ASA I–II, female sex, and the absence of dementia or cognitive impairment were the reference categories.

**Table 8:** Comparison of conventional and Firth estimates for 1-year mortality.

Predictor	Conventional OR	Firth OR	Δβ	Firth/ML OR Ratio
Age, per 10-year increase	2.01	1.93	0.038	0.96
Male sex	2.70	2.61	0.035	0.97
ASA III vs. ASA I–II	3.46	2.83	0.200	0.82
ASA IV vs. ASA I–II	8.18	6.49	0.230	0.79
Dementia/cognitive impairment	3.25	3.08	0.055	0.95
Hemoglobin, per 1 g/dL decrease	1.27	1.26	0.011	0.99
Log-transformed NLR	1.38	1.36	0.012	0.99

Table note: Δβ is calculated as β_ML − β_Firth. An odds ratio close to 1 indicates a limited difference between the conventional and Firth estimates. Values below 1 indicate the shrinkage of the Firth estimate relative to the maximum likelihood estimation.

**Table 9:** Discrimination, overall accuracy, and calibration of conventional and Firth logistic regression models.

Outcome and Model	Apparent AUC	Optimism-Corrected AUC	Apparent Brier Score	Optimism-Corrected Brier Score	Apparent Calibration Slope	Optimism-Corrected Calibration Slope
1-year mortality—conventional logistic	0.807	0.787	0.171	0.182	1.000	0.868
1-year mortality—Firth logistic	0.806	0.786	0.171	0.182	1.056	0.926
30-day mortality—conventional logistic *	—	—	—	—	—	—
30-day mortality—Firth logistic	0.814	—	0.091	—	1.082	—

Table note: Internal validation of the primary 1-year mortality models was conducted using 1000 bootstrap resamples. Optimism-corrected values were calculated by subtracting the average bootstrap optimism from the apparent performance. The conventional logistic model converged in 902 of 1000 bootstrap samples, whereas the Firth model generated finite estimates in all 1000 samples. * The conventional 30-day model showed quasi-complete separation and failed to converge adequately; therefore, the performance measures were not reported for this model. AUC, area under the receiver operating characteristic curve.

## Data Availability

The data are not publicly available because they contain potentially identifiable clinical information. De-identified data may be available from the corresponding author upon reasonable request and subject to institutional and ethical approval.

## Supplementary Materials

The following supporting information can be downloaded at: https://www.mdpi.com/article/10.3390/jcm15187195/s1, File S1: STROBE Statement—Checklist of Items That Should Be Included in Reports of Cohort Studies.
