# Supplementary material for: Aggregation-Sensitive Mortality Associations and Firth Penalized Logistic Regression After Hip Fracture Surgery: A Retrospective Cohort Study
Source: J Clin Med. 2026 Sep 16;15(18):7195. doi: 10.3390/jcm15187195 (PMC13607052; doi:10.3390/jcm15187195)
Supplement: Supplementary file 1 [file jcm-15-07195-s001.zip › jcm-4489261-supplementary.pdf]

Supplementary File S1

STROBE Statement—Checklist of Items That Should Be Included in Reports of Cohort Studies

Manuscript: Aggregation-Sensitive Mortality Associations and Firth Penalized Logistic Regression after Hip Fracture Surgery: A Retrospective Cohort Study

Item No.	STROBE item	Recommendation	Where reported
1	Title and abstract	(a) Indicate the study's design with a commonly used term in the title or abstract.	Title; Abstract, p. 1
1	Title and abstract	(b) Provide in the abstract an informative and balanced summary of what was done and what was found.	Abstract, pp. 1–2
2	Background/rationale	Explain the scientific background and rationale for the investigation being reported.	Introduction, pp. 2–3
3	Objectives	State specific objectives, including any prespecified hypotheses.	Introduction, p. 3
4	Study design	Present key elements of study design early in the paper.	Materials and Methods, Section 2.1, pp. 3–4
5	Setting	Describe the setting, locations, and relevant dates, including periods of recruitment, exposure, follow-up, and data collection.	Section 2.1, pp. 3–4
6	Participants	(a) Give the eligibility criteria and the sources and methods of selection of participants. Describe methods of follow-up.	Section 2.1, pp. 3–4
6	Participants	(b) For matched studies, give matching criteria and number of exposed and unexposed.	Not applicable; this was not a matched cohort study.
7	Variables	Clearly define all outcomes, exposures, predictors, potential confounders, and effect modifiers. Give diagnostic criteria, if applicable.	Sections 2.2–2.3, pp. 4–5
8	Data sources/measurement	For each variable of interest, give sources of data and details of methods of assessment. Describe comparability of assessment methods if there is more than one group.	Sections 2.1–2.3, pp. 3–5
9	Bias	Describe any efforts to address potential sources of bias.	Sections 2.3–2.12, pp. 4–10; Discussion limitations, p. 28
10	Study size	Explain how the study size was arrived at.	Section 2.1, p. 3; final eligible cohort comprised 297 patients from available records.
11	Quantitative variables	Explain how quantitative variables were handled in the analyses. If applicable, describe which groupings were chosen and why.	Sections 2.3, 2.5 and 3.3, pp. 5–6 and 14
12	Statistical methods	(a) Describe all statistical methods, including those used to control for confounding.	Sections 2.4–2.11, pp. 5–10
12	Statistical methods	(b) Describe any methods used to examine subgroups and interactions.	Sections 2.5–2.7, pp. 5–7
12	Statistical methods	(c) Explain how missing data were addressed.	Section 2.12, p. 10
12	Statistical methods	(d) If applicable, explain how loss to follow-up was addressed.	Sections 2.2 and 2.12, pp. 4 and 10
12	Statistical methods	(e) Describe any sensitivity analyses.	A censoring-aware Cox proportional hazards sensitivity analysis was performed using all 297 patients; bootstrap internal validation was also conducted (Sections 2.11 and 2.13; Results Sections 3.5 and 3.8).
13	Participants	(a) Report numbers of individuals at each stage of the study.	Sections 2.1–2.2 and 3.1–3.2; Tables 1–2, pp. 3–4 and 11–13
13	Participants	(b) Give reasons for non-participation at each stage.	Sections 2.1–2.2 and 2.12, pp. 3–4 and 10
13	Participants	(c) Consider use of a flow diagram.	Not included; participant numbers and outcome-specific exclusions are reported in text and Table 2.
14	Descriptive data	(a) Give characteristics of study participants and information on exposures and potential confounders.	Section 3.1 and Table 1, pp. 11–13
14	Descriptive data	(b) Indicate number of participants with missing data for each variable of interest.	Section 2.12, p. 10; no missing predictor values in primary models.
14	Descriptive data	(c) Summarize follow-up time.	Sections 2.2 and 3.2; Table 2, pp. 4 and

Item No.	STROBE item	Recommendation	Where reported
			13
15	Outcome data	Report numbers of outcome events or summary measures over time.	Section 3.2 and Table 2, p. 13
16	Main results	(a) Give unadjusted and, if applicable, confounder-adjusted estimates and their precision. Make clear which confounders were adjusted for and why.	Sections 3.3–3.7; Tables 3–8, pp. 14–22
16	Main results	(b) Report category boundaries when continuous variables were categorized.	Section 3.3, p. 14
16	Main results	(c) If relevant, consider translating estimates of relative risk into absolute risk for a meaningful time period.	Fixed-time mortality proportions are reported in Section 3.2 and Table 2, p. 13.
17	Other analyses	Report other analyses performed, such as subgroup analyses, interactions, and sensitivity analyses.	Sections 3.3–3.5 and 3.8; Tables 3–4 and 9.
18	Key results	Summarize key results with reference to study objectives.	Discussion, opening paragraph, pp. 23–24
19	Limitations	Discuss limitations of the study, taking into account sources of potential bias or imprecision.	Discussion, p. 28
20	Interpretation	Give a cautious overall interpretation of results considering objectives, limitations, multiplicity of analyses, and evidence from similar studies.	Discussion, pp. 24–28
21	Generalisability	Discuss the generalisability (external validity) of the study results.	Discussion limitations, p. 28
22	Funding	Give the source of funding and the role of the funders.	Funding statement, p. 29; no external funding.
